# Picture Me Drinking: Alcohol-Related Posts by Instagram Influencers Popular Among Adolescents and Young Adults

**DOI:** 10.3389/fpsyg.2019.02991

**Published:** 2020-01-22

**Authors:** Hanneke Hendriks, Danii Wilmsen, Wim van Dalen, Winifred A. Gebhardt

**Affiliations:** ^1^Amsterdam School of Communication Research, University of Amsterdam, Amsterdam, Netherlands; ^2^Dutch Institute for Alcohol Policy STAP, Utrecht, Netherlands; ^3^European Centre for Monitoring Alcohol Marketing, Utrecht, Netherlands; ^4^Institute of Psychology, Health, Medical and Neuropsychology Unit, Leiden University, Leiden, Netherlands

**Keywords:** social media, influencers, alcohol consumption, adolescents and young adults, alcohol brands, sponsorship disclosure

## Abstract

Research has shown that young people post a lot of alcohol-related posts (i.e., alcoholposts) on social media and these posts have been shown to increase drinking behaviors. Because social influencers (i.e., individuals with the potential to influence large audiences on social media) may have a strong influence on young people, it is important to know whether and how often they post about alcohol. Furthermore, because by using influencers alcohol brands may have found a way to circumvent regulations that prohibit advertising for minors, it is important to understand whether alcohol brands are visible in influencers’ posts and whether influencers use disclosures (e.g., “#ad”) to notify viewers. In a content analysis of Instagram posts of 178 popular influencers, we investigated: (1) how many and how often influencers post about alcohol, (2) what type of influencers post about alcohol, (3) what the characteristics of influencers’ alcoholposts are, and (4) to what extent these alcoholposts are commercialized (e.g., by showing brands and sponsorship disclosures). Results showed four main findings. (1) The majority of influencers (i.e., 63.5%) posted about alcohol recently. (2) Alcoholposts were positive, showed a social context, and were mostly posted by *lifestyle influencers*. (3) Although a fair amount of alcoholposts (19.5%) showed a clear alcohol brand, only a few of these posts disclosed this as an advertisement, and even fewer gave an educational slogan (i.e., “#no18noalcohol”). (4) Posts with sponsorship disclosures yielded fewer likes and comments than posts without such disclosures. A *post hoc* additional study that focused solely on minors confirmed these conclusions. These findings suggest that there is a lot to be concerned about in this context, especially since many minors can be exposed to influencers’ alcoholposts, potentially leading to increased drinking among this vulnerable age group. We therefore advice future researchers to further investigate this topic, and propose that legislation for alcohol advertising needs to be adjusted to account for the context of social networking sites.

## Introduction

Frequent and excessive alcohol use are important problems in today’s Western society, especially among adolescents and young adults. Young people start drinking quite early on. For example, in the Netherlands, most adolescents drink their first alcohol consumption when they are 14 or 15 years of age, and almost half of all 16 year olds have engaged in binge drinking at least once (i.e., more than 4/5 drinks on one occasion), even though the legal purchasing age in the Netherlands is 18 years (Van Dorsselaer et al., 2016; Nationale Drug Monitor, 2017; Stevens et al., 2017). After leaving high school, drinking patterns tend to intensify: a study in the United Kingdom, for example, showed that almost two-thirds of college students have a hazardous alcohol consumption score on the AUDIT scale (Davoren et al., 2016). The negative consequences of alcohol use are well-documented, and include increased chances of vandalism, accidents, abuse, and brain and liver damage (Rehm et al., 2003; Hughes et al., 2008). The earlier young people start drinking alcohol, the more likely are they to develop alcohol dependence and suffer from serious accidents (Hingson and Zha, 2009). Moreover, the negative effects of alcohol on brain development are especially pronounced during adolescence (Silveri, 2012). In line with this, the World Health Organization [WHO] (2006, p.14) therefore advises to “keep children alcohol free and delay the onset of drinking.” For this purpose, it is vital to gain insight in factors that might influence alcohol use among adolescents. The current study aims to do this by investigating how Instagram influencers, who are popular among minors (e.g., Ofcom, 2017), communicate about alcohol and thereby potentially influence alcohol use among their young audiences.

### Alcoholposts on Social Media

Since the rise of new technologies, young people increasingly spend time “online.” 95% of teens have access to a smartphone and almost half of them indicate “to be online almost constantly” (Anderson and Jiang, 2018). One of the most prominent pastimes of adolescents is to engage with others on social media, such as Youtube, Facebook, and Snapchat, with Instagram presently being the most popular platform among adolescents (Anderson and Jiang, 2018). Studies show that alcohol is a very recurring theme on social networking sites. Although reported percentages vary (i.e., from 36 to 96%), young people often place alcohol-related posts (henceforth: alcoholposts) on social networking sites (Moreno et al., 2010, 2012; Hendriks et al., 2017; Curtis et al., 2018). Examples of such posts are dinner pictures with drinks on the table, group party pictures in which people are holding alcoholic drinks, or close-ups of cocktail glasses (Beullens and Schepers, 2013; Hendriks et al., 2018a). Studies have revealed that these posts are often social in nature (i.e., showing groups of people) and portray positive associations with alcohol (i.e., laughing people; Hendriks et al., 2018b).

These positive and social posts are likely to enhance the perception that drinking alcohol is normal and “fun,” and consequently may encourage alcohol use. This is in line with classical theories, such as the two-step flow theory and the diffusion of innovations theory (Lazarsfeld et al., 1944; Katz, 1957; Rogers, 1983), suggesting that messages are spread through processes of interpersonal communication. Furthermore, this is in line with both social norms theory and social learning theory (Bandura and Walters, 1977; Perkins and Berkowitz, 1986; Berkowitz, 2004). Social norms theory describes that behavior is based on people’s perceptions of how others behave and what they approve of, and similarly social learning theory suggests that behavior can be learned from observing others. Both theories would seem to suggest that seeing alcoholposts on social media leads to the perception that others are also doing it (i.e., descriptive norms) and approve of it (i.e., injunctive norms; Cialdini and Trost, 1999). Although the number of longitudinal and experimental studies looking into the effects of alcoholposts on alcohol use are limited, there are some cross-sectional studies that indeed show a strong relationship between the prevalence of alcoholposts on social media and alcohol (ab)use (Boyle et al., 2016; Geusens and Beullens, 2016; Curtis et al., 2018), as well as alcohol-related problems, such as alcohol-induced fights (Thompson and Romo, 2016). Importantly, studies confirm that not only posting, but also seeing, alcoholposts can increase alcohol use (Geusens et al., 2019).

### Influential Others: The Case of Influencers

Alcoholposts can thus increase alcohol use among young people. However, it is highly likely that some people in their social context are more influential in this respect than others. In the present study, we focus on the influential impact of celebrities. The fact that celebrities have persuasive power has already been used in many advertising contexts, resulting in what is now known as celebrity endorsement (Erdogan, 1999). A recent meta-analysis showed that products endorsed by a celebrity lead to more positive attitudes toward the product and higher buying intentions, especially if the “type” of celebrity matches well with associative characteristics of the product (Knoll and Matthes, 2017). Both the meaning-transfer model (McCracken, 1986) and the associative learning theory (Collins and Loftus, 1975) explain such influence of celebrities by suggesting that positive characteristics associated with the celebrity can be transferred to the product. Celebrities are often seen as attractive, trustworthy, and an expert. While adults seem to be quite able to distinguish between these aspects and are often less influenced by merely the attractiveness of the source, teenagers find this harder and are therefore more influenced by attractive celebrities who are not necessarily an expert or trustworthy (Lord and Putrevu, 2009). This vulnerability of young people for celebrity endorsement has also been shown in the context of alcohol consumption. For example, teens (13–17 years old) are more likely to recall exposure to online alcohol advertisements and pictures including celebrities than adults (Jernigan et al., 2017).

Since the rise of social media, a new type of celebrity has emerged: the so-called social influencer. Although a general definition does not yet exist, a social influencer refers to an individual who can influence others and who builds their audience through social media (Gross and Wangenheim, 2018). Although in theory everyone could be or become a social influencer, often a number of followers are assumed for influential influencers. For example, a minimum of 5,000–10,000 followers is often assumed for macro- or mid-tier influencers (Agrawal, 2019). Social influencers may be especially persuasive because they are more similar and approachable for young people than celebrities in traditional media, thereby creating stronger feelings of intimacy (Berryman and Kavka, 2017). Research suggests that more similar and proximate others have a stronger effect on norm transitions than those who are viewed as more distinct and distant. For example, Van den Putte et al. (2011) showed that conversations with similar peers have a stronger normative impact than dissimilar ones, and Boer and Westhoff (2006) found that stronger connections (e.g., when people are close, friends) lead to more effects of communicated norms than weaker connections (i.e., when people are distant, strangers). Social influencers are not considered to be strangers but rather experienced as people with whom followers feel closely connected (Berryman and Kavka, 2017). The potential of social influencers to affect their young audiences has been recognized by companies worldwide, as can be seen by the fact that 63% of US companies have increased their influencer marketing budget in 2017 (Gross and Wangenheim, 2018).

### Influencers and Alcoholposts

Although evidence exists of peers sharing alcohol-related content, it is not clear how often influencers who are popular among minors post about alcohol. Given the potential strong impact influencers might have, it is important to understand their portrayal of alcoholposts on social media. In addition, influencers are very popular among children and many influencers have minors as followers (Ofcom, 2017). In fact, it has been shown that more than one-third of 14–17 year olds look deliberatively for influencer accounts when they are looking for product information (Thomasius, 2018). By seeing alcoholposts from influencers, these minors might be encouraged to start drinking, or if they already drink, to consume more alcohol. In the context of unhealthy foods, recent evidence indeed suggests that children’s exposure to Instagram influencers eating unhealthy foods increased unhealthy snacking later on (Coates et al., 2019). A similar undesirable effect of influencers posting about alcohol is highly plausible, making it important to gain knowledge on the alcoholposts that are being posted by social influencers.

The need to gain insight in influencers’ display of alcoholposts is increased even further because in many countries regulations state that alcohol advertising is not to target minors. For example, in the Netherlands, there is no alcohol advertising on traditional media when children are awake (i.e., between 6 AM and 9 PM) and in the United Kingdom and the United States, alcohol ads can only be shown when the majority of the audience is older than 18/21 years (Jackson et al., 2000; Global Advertising Lawyers Alliance, 2011). Banning alcohol advertising directed at minors is also reflected in the ethical principles for alcohol policy of the World Health Organization [WHO] (2006, p. 2), in which it is stated that “All children and adolescents have the right to grow up in an environment protected from the negative consequences of alcohol consumption and (…) from the promotion of alcoholic beverages.” However, by having influencers advertise for alcohol products, alcohol brands may have found a way to circumvent these regulations. This is even more disturbing because research shows that influencer marketing elicits less resistance to the ad message than traditional advertisements do (de Vries et al., 2012). It is therefore relevant to know how often influencers show alcohol brands in their posts, and to what extent they disclose this form of advertising (i.e., by indicating this is sponsored content, and/or by giving an educational slogan such as “No 18, no alcohol,” as advised by the Dutch Foundation for Responsible Alcohol Consumption).

### The Present Study

The main goal of the current study is to investigate influencers’ alcoholposts on Instagram. We conducted a content analysis of alcoholposts placed by the most popular Instagram influencers among adolescents and young adults in the Netherlands. Four research questions were addressed: (RQ1) How many and how often do influencers post about alcohol? (RQ2) What “type” of influencers post about alcohol (e.g., *beauty* influencers versus *lifestyle* influencers)? (RQ3) What are the characteristics of influencers’ alcoholposts (e.g., do they show a positive and social context)? (RQ4) In what way are these alcoholposts commercialized (i.e., are brands visible, is there a sponsorship disclosure, and is there an educational slogan?) and are these commercial aspects related to likes and comments?

In a *post hoc* additional study among minors, we investigated whether similar results would be obtained regarding RQ1 and RQ4.

## Materials and Methods

### Content of Coding – Influencers

We aimed to study popular influencers, i.e., that have many followers and are often liked by adolescents and young adults. Therefore, we first conducted a pilot study among young people (i.e., students; *N* = 362, 18–25 years) in which we asked participants to list their top 3 favorite Instagram influencers. We chose Instagram, because it is considered to be the most important social network for influencers and the most popular social networking site for adolescents (Hashoff, 2017). Based on the initial pilot study, we identified 281 unique “influencers.” Next, we looked at the number of followers and included all influencers with more than 10,000 followers. Most sources agree that micro influencers range from 5,000/10,000 to 20,000 followers and that meso influencers have 20,000 followers or more (Domingues Aguiar and van Reijmersdal, 2018; Agrawal, 2019; Boerman, 2019). We chose to use 10,000 followers as a minimum to be able to include “micro,” “macro,” “meso,” and “mega” influencers. As a result, we also avoided including “nano” accounts that were not owned by professional influencers. Furthermore, we excluded some accounts that were not real influencer accounts (i.e., meme accounts or accounts in which no person was visible) or accounts that were set to private. Based on these criteria, a total of 178 influencers emerged that were to be coded. These 178 influencers had on average 646,323 followers (*SD* = 1,802,009, range = 11,200–16,500,000), and the majority of the influencers were female (117 women; 59 men).

### Content Analysis

#### Codebook Development and Procedure

The codebook was based on a codebook that has been used to code alcoholposts on social media by Hendriks et al. (2018a, b) and adjusted to fit the topic of influencers. The first coder (between June 27 and July 2, 2019) examined all influencer’ profiles, and the most recent 100 posts were viewed to observe if they could be regarded as an alcoholpost. In line with Hendriks et al. (2018a, b), an alcoholpost was defined as “a post about alcohol, or in which alcohol is visible.”

##### Occurrence and frequency

The coder thus identified whether there were alcoholposts visible (i.e., occurrence: no/yes), and how many were identified in the last 100 posts (i.e., frequency). The first coder coded all 17,800 posts for the presence of alcoholposts. This was done during a period of several days to reduce coder fatigue. The coder coded for each alcoholpost specific information (see below). Next, a second coder also coded 10% of all alcoholposts to assess coder reliability, which was considered acceptable/good. That is, reliability was good for characteristics of alcoholposts (K’s Alpha_social_ = 0.95, and K’s Alpha_typedrink_ = 0.73), disclosures (K’s Alpha = 0.84) and slogans (K’s Alpha = 1.00), and acceptable for brands being visible (K’s Alpha = 0.69). The information retrieved and coded involved the following.

##### Characteristics of the alcoholpost

In line with Hendriks et al. (2018b), it was indicated whether the alcoholpost was social (showing no; 1; or 2 + people) and whether the context of alcohol was positive (e.g., showing laughing people or positive consequences of alcohol use [having fun]) or negative (e.g., showing frowning people or negative consequences of alcohol use [a hangover]). Furthermore, it was coded what type of alcoholic drink was shown (e.g., beer; wine; spirits; cocktails; 0% alcohol) and how many likes and comments a post received.

##### Brands visible

It was indicated whether the alcoholpost clearly (i.e., full brand name, recognizable logo, or brand name in header or tag visible) showed an alcohol brand, and if so which one.

##### Sponsorship disclosures

It was coded whether such a brand post had a disclosure of sponsored content (no/yes) and if so, what this disclosure involved (e.g., “#ad”).

##### Educational slogan

It was coded whether such a brand post had an educational slogan (i.e., “no 18, no alcohol”), that is advised by the Dutch Foundation for Responsible Alcohol Consumption.

Furthermore, for each influencer, the following information was coded.

##### Type of influencer

Based on Influencity (2018), Forsey (2019), and Lou and Yuan (2019), six types of influencers were discerned. An influencer was coded to be a: (1) *Beauty* influencer when mainly posts explaining how beauty products work (e.g., tutorials, materials, and end results) were displayed, (2) *Fashion* influencer when the account mainly involved fashion and clothing attributes, (3) *Food* influencer when mainly posts on food and drinks, including recipes were shown, (4) *Lifestyle* influencer when mostly photos of the influencer’s daily life were depicted (e.g., parties, dinners, beach, and street photos), (5) *Sport and fitness* influencer when the account focused on physical activity and visible sport outings (including sport supplements, sports clothing, and looking fit), or (6) *Travel* influencer when pictures of different travel locations in different countries were posted, and when the account resembled a travel blog.

##### Number of followers

Coders also noted the number of followers at the time of coding.

## Results

### How Many and How Often Do Influencers Post About Alcohol?

We coded the first 100 posts of 178 influencers, leading to a total number of posts of 17,800. In total 384 alcoholposts (2.2%) were posted. Focusing on the influencers, 113 out of 178 (63.5% percent) influencers had at least one alcoholpost among the last 100 posts with an average of 3.40 (*SD* = 3.18) alcoholposts. Among these 113 influencers, 43 influencers (38.1%) posted one alcoholpost among the last 100 posts, 32 influencers (28.3%) posted two or three alcoholposts, 18 influencers (15.9%) posted four or five alcoholposts, seven influencers (6.2%) posted six or seven alcoholposts, six influencers (5.3%) posted eight or nine alcoholposts, three influencers (2.7%) posted 10 or 11 alcoholposts, three influencers (2.7%) posted 13 or 15 alcoholposts, and one influencer (0.9%) posted 16 alcoholposts (see also Table 1).

**TABLE 1:** Influencers’ posting of alcoholposts.

**# Alcoholposts**	**# Influencers**	% Influencers*	Examples influencers**	**# Followers**
16	1	0.88	Lterveld	33,100
15	1	0.88	Lilkleine	1,600,000
13	2	1.77	Koenkardashian; Leoalkemade	137,000; 31,800
11	2	1.77	Hollybrood; Dailyrebel	181,000; 23,300
10	1	0.88	Geraldine_kemper	568,000
9	4	3.54	Kajstypetjes; Sylvana; Alexx.mass	513,000; 181,000; 124,000
8	2	1.77	Monicageuze; Mariekeelsinga	1,100,000; 279,000
7	1	0.88	Phalerieau	74,300
6	6	5.31	Nicolettevandam1; Ninawarink; Koenweijland	704,000; 391,000; 123,000
5	6	5.31	Fredvanleer; Sophiemilzink; Claartjerose	693,000; 436,000; 377,000
4	12	10.62	Giel; Alibspec; beautygloss	950,000; 749,000; 579,000
3	16	14.16	Sneijder10official; Boef; K2im	2,100,000; 1,200,000; 484,000
2	16	14.16	Dee; Andrehazes; Nigeldejong	1,300,000; 726,000; 520,000
1	43	38.05	Nikkietutorials; Memphisdepay; Maxverstappen1	12,300,000; 7,000,000; 2,200,000
0	65	-	Martingarrix; Doutzen; Romeestrijd	16,500,000; 6,100,000; 5,500,000

*Percentage among influencers posting alcoholposts (*N* = 113). **The influencers with the highest number of followers within that category are shown.

### What Type of Influencers Post About Alcohol?

Most influencers were coded as *lifestyle* influencers (*n* = 128, 71.9%), *fashion* influencers (*n* = 19, 10.7%), or as *sport and fitness* influencers (*n* = 19, 10.7%). The other types, i.e., *beauty*, *food*, and *travel*, were not coded often (*n* was, respectively, 2; 5; 5). Therefore, the subsequent analysis only used the influencer types that were coded as *lifestyle*, *fashion*, or *sport and fitness*.

To explore whether the number of alcoholposts depended on the type of influencer, an ANOVA was conducted with influencer type as independent variable and number of alcoholposts as dependent variable. The analysis revealed a significant effect of influencer type, *F*(2,163) = 4.83, *p* = 0.009. That is, we found that *lifestyle* influencers posted on average 2.60 alcoholposts (*SD* = 3.37); significantly more than *fashion* (*M* = 0.89, *SD* = 1.15) or *sport and fitness* influencers (*M* = 0.84, *SD* = 1.07).

### What Are the Characteristics of Influencers’ Alcoholposts?

#### Context and Social

The following analyses focused only on the alcoholposts (in total 384 alcoholposts were posted). In line with Hendriks et al. (2018b), all alcoholposts were coded to have a positive context (100%). In addition, most posts were social (i.e., had more than one person visible): i.e., 230 posts (59.9%) showed two or more people, 130 posts (35.2%) showed one person, and 19 posts (4.9%) showed no persons visible.

#### Type of Alcohol

Most alcoholposts showed wine (*n* = 217, 56.5%), followed by beer (*n* = 79, 20.6%), cocktails (*n* = 55, 14.3%), spirits (*n* = 31, 8.1%), and 0% alcohol (*n* = 2, 0.5%).

#### Likes and Comments

The number of likes and comments varied enormously between posts and influencers. The minimum number of likes for an alcoholpost was 119 and the maximum number of likes for an alcoholpost was 546,232 (*M* = 24,057, *SD* = 46,183). The minimum number of comments on an alcoholpost was 0 and the maximum number of comments on an alcoholpost was 13,992 (*M* = 281.24, *SD* = 975.92).

### How Commercialized Are Influencers’ Alcoholposts?

#### Brands Visibility

Seventy-five out of 384 alcoholposts (19.5%) clearly showed a brand. Out of these branded posts, 50 posts showed a clear alcohol brand in the photo, 10 posts only had the brand name in the header or hashtag, and 15 posts had the brand in both the picture and the text. In all branded posts, 30 different brands were featured in total. The top 10 most often shown alcohol brands can be found in Table 2.

**TABLE 2:** Top 10 alcohol brands visible in influencers’ alcoholposts.

**Rank**	**Brand**	**# Posts**	**% Branded posts**
1	Moët	10.00	33.33
2	Heineken	9.00	12.00
3	Desperados	7.00	9.33
4	Absolut Vodka	4.00	5.33
5	Amstel	4.00	5.33
6	Original Peachtree	4.00	5.33
7	Brouwerij ’t Ij	3.00	4.00
8	Canei	3.00	4.00
9	Jagermeister	3.00	4.00
10	Ketel One Vodka	3.00	4.00

#### Sponsorship Disclosures

Out of the 75 branded alcoholposts, only 25 (33.3%) showed an indication of sponsorship disclosure. Most disclosures (*n* = 19) had the form of a hashtag (e.g., “#ad”), although others used a caption, tagged the brand, or mentioned “paid partnership with…” (*n* = 6).

#### Educational Slogan

Out of these 25 branded alcoholposts with a sponsorship disclosure, only eight (32%) used the educational slogan “geen18geenalcohol” (translated: “no 18 no alcohol”) as advised by the Dutch Foundation for Responsible Alcohol Consumption. Table 3 shows four examples of alcoholposts that we have coded (i.e., no brand; brand no disclosure; brand disclosure, brand disclosure slogan).

**TABLE 3:** Four examples of alcoholposts.

**Type of alcoholpost**			
**No alcohol brand visible**	**Alcohol brand, no disclosure**	**Alcohol brand, disclosure, no slogan**	**Alcohol brand, disclosure, slogan**
https://www.instagram.com/nikkietutorials/p/Bqn0DAWjpxb/?hl=nl	https://www.instagram.com/p/B0IcRPLAXMr/	https://www.instagram.com/p/Byu635dCAEv/	https://www.instagram.com/p/BzKbyd0IiGr/?hl=nl

**Links are shown instead of pictures due to copyright reasons.**

### Relationships Between Commercialized Aspects and Likes and Comments

We also explored whether these commercialized characteristics of the alcoholposts were related to the likes and comments of the posts. Before conducting analyses, both comments and likes were log transformed because the variables were heavily skewed and high in kurtosis. Whether a brand was visible or not did not significantly influence the number of likes or comments (all *F* < 1.59, all *p* > 0.208). However, the presence of a disclosure did influence the number of likes and comments. That is, two ANOVAs with disclosure presence (no/yes) as independent variable and number of likes and comments as dependent variables revealed that alcoholposts with a sponsorship disclosure resulted in significantly fewer likes (*M* = 3.50, *SD* = 0.45) and comments (*M* = 1.53, *SD* = 0.38) than when no such disclosure was present (*M_likes_* = 3.93, *SD*_*likes*_ = 0.79; *M_comments_* = 1.95, *SD*_*comments*_ = 0.91), *F*_*likes*_(1,73) = 6.25, *p_likes_* = 0.015, η^2^*_*likes*_* = 0.079; *F*_*comments*_(1,73) = 4.88, *p_comments_* = 0.030, η^2^*_*comments*_* = 0.063. Educational slogans did not influence likes and comments (all *F* < 1.25, all *p* > 0.267), although this could be due to the low number of posts with slogans (*n* = 8).

### The Case of Minors: An Additional Study^1^

The abovementioned results were based on influencers mentioned in a pilot study which was conducted among students aged 18–25 years. To explore the extent to which the results described above apply to minors, we conducted another study in which we solely focused on minors (*N* = 121, *M_age_* = 16.63, *SD_age_* = 0.49, range*_*age*_* = 16–17 years, 69 girls and 52 boys). In this study, we asked whether these minors followed the 113 influencers mentioned in the first study who posted alcoholposts. Minors indicated that they followed almost all of these influencers (i.e., 105 influencers were mentioned at least once; only eight influencers were not followed at all). Furthermore, we asked the minors to list their top 3 Instagram influencers. Forty-eight influencers corresponded with those mentioned in the first study. To investigate whether the same conclusions could be drawn based on the influencers mentioned by minors, we again conducted the most important analyses mentioned in the previous study focusing solely on these 48 influencers.

Analyses showed that 32 out of these 48 influencers (66.7%) had at least one alcoholpost on their profile. The mean number of alcoholposts was 2.29 (*SD* = 3.11, range 1–15). In total these 32 influencers posted 109 alcoholposts. Out of these 109 posts, 22 (20.2%) were branded posts. Among these 22 posts, only four mentioned a disclosure and three had an educational slogan (18.2; 13.6%). These percentages for influencers mentioned by minors are very similar to those percentages for influencers mentioned by students (which were, respectively, 63.5%; 19.5%; 33.3–32%).

Lastly, we asked an additional question in the second pilot study (i.e., “do you follow influencers who sometimes post alcoholposts? [and if so, “how often do you see such posts by influencers”]); 57 minors (47.1%) indicated to see alcoholposts by influencers, and the majority of these minors (i.e., *n* = 47; 82.5%) indicated to see influencers’ alcoholposts at least two to four times a month, and many (*n* = 24; 42.1%) said they saw influencers’ alcoholposts at least two to six a week. Taking these findings together, we believe that the results apply to both young adults as well as minors.

## Discussion

Although research has shown that alcoholposts from peers on social media can lead to increased drinking among adolescents (e.g., Boyle et al., 2016; Curtis et al., 2018), no studies to date have explored whether and how social influencers, who are likely to have a strong impact on minors (e.g., Berryman and Kavka, 2017), communicate about alcohol. The goal of this study was therefore to investigate influencers’ alcoholposts on one of the most popular social media, i.e., Instagram. Four main results were found in a first study among students (18–25 years). First, the majority of influencers (i.e., 63.5%) posted about alcohol recently. Second, these alcoholposts were mostly posted by *lifestyle influencers*, were positive, and showed a social context. Third, although a fair amount of (19.5%) alcoholposts clearly showed an alcohol brand, only a few of these posts disclosed this content as an advertisement, and even fewer gave an educational slogan. Fourth, posts with sponsorship disclosures had fewer likes and comments than posts without such disclosures. The first and fourth findings were confirmed in a second study among minors.

The first and foremost finding was that a large majority (i.e., 63.5%) of the influencers had at least one alcoholpost among their most recent 100 posts. Furthermore, although on average these influencers posted three alcoholposts, there were some influencers who posted 15–16 alcoholposts. Considering the fact that the influencers had on average approximately 650,000 followers, and that some influencers posting alcoholposts had even more than 12 million followers (e.g., *Nikkietutorials*), this shows that youths nowadays are massively exposed to influencers’ alcoholposts. This massive exposure to influencers’ alcoholposts is very worrisome, especially because influencers are new kinds of celebrities that young people nowadays frequently aspire to become themselves (Berryman and Kavka, 2017).

Another very relevant issue here is age. Influencers are part of a new trend in social media, and it seems that especially adolescents are Instagram users and follow influencers most actively (Ofcom, 2017; Thomasius, 2018). Although it is hard to prove that followers of a certain influencer are underage (i.e., users’ age is not directly visible on Instagram), there are some cumbersome methods to infer the age of some of the followers. For example, Ruijs (2018) randomly selected 200 followers of several influencers, visited their profile, and if possible, assessed whether they were underage (e.g., by looking at “happy birthday posts”). He found that several influencers included in this study had quite a large number of minors among their followers. For example, 18.5% of the followers of *Geraldine Kemper* were minors (200 random picks: 37 minors, 88 above 18 years, 75 age not possible to discern [e.g., private profile]). Among de *Broer van Roos* followers, 23.9% were minors (109 random picks: 26 minors, 40 above 18 years, 43 age not possible to discern). Furthermore, *Lizekorpie* had 20% minors among her followers (150 random picks: 30 minors, 65 above 18 years, 55 age not possible to discern). As can be seen in the results, these influencers posted, respectively, 10, 4, and 6 alcoholposts, and they have many followers (i.e., 568,000; 290,000; 53,500 followers) thereby highlighting that these alcoholposts were potentially shown to a very large group of minors (i.e., 105,080; 69,310; 10,700 minors). Furthermore, our second study quite clearly shows that many minors are exposed to alcoholposts by influencers, and that for many of them this happens on a regular basis (e.g., once a week). Seeing such alcoholposts may make minors more likely to start drinking, or if they already do, drink more alcohol more often. As argued earlier, alcohol use among minors can lead to a high likelihood of alcohol dependence also later in life, serious accidents, and negative effects on brain development (e.g., Silveri, 2012). This stresses the importance of addressing the fact that influencers’ alcoholposts are shown to minors.

The second finding related to the content of the alcoholposts. We found that mainly *lifestyle* influencers posted about alcohol, and that they solely do this in a positive way (e.g., by showing laughing people), and often show a social context (i.e., showing one or more persons). This is very much in line with earlier content analyses of peers’ alcoholposts on social media, in which it has been stressed that alcoholposts are positive and social in nature (Hendriks et al., 2018b). In light of social norms theory and social learning theory (Bandura and Walters, 1977; Perkins and Berkowitz, 1986; Berkowitz, 2004), this makes it very likely that influencers’ alcoholposts increase alcohol use, because not only do they show positive instead of negative associations with alcohol use (i.e., leading to positive alcohol-related beliefs), they also show that many others engage in and approve of alcohol use (i.e., leading to positive alcohol-related norms). It is interesting that these seemingly inherent characteristics of alcoholposts on social media are similar for peer-to-peer posts as for influencers’ posts. This may suggest that posts by peers are actually quite similar to posts by influencers, thereby increasing feelings of similarity between the “normal” social media users and those of celebrity status.

Third, we also looked at how commercialized influencers’ alcoholposts were. Findings showed that a fair share (i.e., 19.5%) of alcoholposts showed a clear alcohol brand. However, only one-third of these branded posts showed a sponsorship disclosure (i.e., “#ad”), and even fewer had the desirable educational slogan “no 18, no alcohol.” Although we cannot be certain that the two-thirds of branded posts without a disclosure were actually disguised commercials, we can say that some of the posts (e.g., the *Bintang* post in Table 3) showed the brand in a very prominent way. Considering the fact that most of these influencers have many followers and earn a lot of money for a single advertised post, it seems hard to believe that they would advertise for these brands for free. If it is indeed the case that some of these influencers were paid to advertise for an alcohol brand, it is questionable that they advertise for alcohol-related products while not being transparent about this. Furthermore, as argued, it is highly likely that there are many minors being exposed to this branded content. If it is indeed the case that influencers advertise for alcohol brands (as might be inferred indirectly from the 75 branded alcoholposts, but can be directly inferred from the 25 posts with sponsorship disclosures), then this suggests that the alcohol industry has found a way to circumvent legislation and reach minors (Global Advertising Lawyers Alliance, 2011). Although some suggest that internet age filters or entry pages are good ways of limiting exposure to underage alcohol marketing, evidence suggests that these filters do not work effectively and still allow minors to see alcohol ads (Jones et al., 2014). This stresses the need for new legislation that also incorporates the complicated new world of social media.

A last interesting finding was that disclosures were related to likes and comments. That is, we found that if influencers disclosed that they advertised for an alcohol brand this was related to fewer likes and comments than when they did not give such a disclosure (but still showed the brand). This is in line with studies that suggest that people can become negative toward the origin of a message (e.g., the influencer) if they see a sponsorship disclosure (e.g., Boerman et al., 2015). This touches upon some practical implications, because for influencers it can be difficult how to communicate with their followers about branded content. On the one hand, influencers claim they want to be transparent with their audience (e.g., in the Netherlands influencers have drawn up a *Social Code* in which they advise how to communicate openly); however, they also do not want to lose popularity because followers are their main source of income. Their dilemma is thus quite understandable. A potential solution would be to stimulate (e.g., potentially reinforced by Instagram itself) every influencer who is being paid in one way or another for a post to disclose this clearly in that post. This is in line with new legislation in some countries (e.g., Germany; Knitter, 2019) in which it is obligatory for all influencers to disclose a post as advertising if they have received a form of compensation for it. Applying such legislation may be useful and clear in other countries as well, because if every influencer is required to behave in the same way, the potential loss of popularity is evenly divided among all influencers.

### Limitations and Future Research

This study is not without limitations. First, our first study was conducted among older adolescents and young adults. Therefore, the influencers we analyzed might differ slightly from those influencers popular among minors. However, as already argued, based on a previous pilot study (Ruijs, 2018), we can be quite certain that several of the influencers we studied have a fair share of minors among their followers. Furthermore, based on the second study that we conducted among minors, we were able to make somewhat stronger claims about the generalizability of the results to the context of minors’ exposure to alcoholposts of influencers. Despite this evidence, we still advise future researchers to investigate in depth what the underage audience is of influencers who post alcoholposts. Although this is a challenging and cumbersome task, this is essential to understand the full exposure of minors to alcohol content posted by influencers.

A second limitation is that we looked at the content of the influencers’ profiles, and did not link this to the responses (e.g., drinking behavior) of teens directly. We argue that our study is an essential first step, because to understand the effects of a certain phenomenon, it is important to first gain insight into the extent of the problem. Now this step has been taken, a next phase is to directly examine the effects of influencers’ alcoholposts on drinking among adolescents and young adults. Although we expect these effects to be even stronger (and thus more undesirable) than posts by peers, future research needs to ascertain whether this is indeed the case.

## Conclusion

This study is the first to illustrate how influencers communicate about alcohol on Instagram. We found that the majority of influencers posted about alcohol. Furthermore, although quite a few posts showed alcohol brands, only a couple of these posts disclosed this content as an advertisement, and even fewer gave an educational slogan. There is thus a lot to be concerned about in this context, especially since many minors can be exposed to such alcoholposts, potentially leading to increased drinking among this vulnerable age group. We therefore advice future researchers to further explore this issue, and suggest that adjustments in legislation for alcohol advertisements are necessary to effectively account for the context of social media.

## Data Availability Statement

The datasets generated for this study are available on request to the corresponding author.

## Ethics Statement

The studies involving human participants were reviewed and approved by the Leiden University (CEP19-0226/133) and the University of Amsterdam (2019-PC-11372). The patients/participants provided their written informed consent to participate in this study.

## Author Contributions

HH collected the data, analyzed the results, and wrote the first and second draft. DW collected the data and provided feedback on the first draft. WD and WG provided feedback on the first draft.

## Conflict of Interest

The authors declare that the research was conducted in the absence of any commercial or financial relationships that could be construed as a potential conflict of interest.

## Footnotes

1 This follow-up study was a result of the review process of an earlier version of this manuscript.

## References

[B1] Agrawal H. (2019). *The Rise of Nano Influencers: How Many Followers do You Need to Become an Instagram Influencer?* Available at: https:// hypeauditor.com/blog/the-rise-of-nano-influencers-how-many-followers-do- you-need-to-become-an-instagram-influencer/ (accessed August 28, 2019).

[B2] Anderson M. Jiang J. (2018). Teens, social media & technology 2018. *Pew Res. Center* 31:2018.

[B3] Bandura A. Walters R. H. (1977). *Social Learning Theory.* Englewood Cliffs, NJ: Prentice-hall.

[B4] Berkowitz A. D. (2004). *The Social Norms Approach: Theory, Research, and Annotated Bibliography.* Available at: https://tobh.pw/h_xu_pog_ruby.pdf

[B5] Berryman R. Kavka M. (2017). ‘I guess a lot of people see me as a big sister or a friend’: the role of intimacy in the celebrification of beauty vloggers. *J. Gend. Stud.* 26 307–320. 10.1080/09589236.2017.1288611

[B6] Beullens K. Schepers A. (2013). Display of alcohol use on Facebook: a content analysis. *Cyberpsychol. Behav. Soc. Netw.* 16 497–503. 10.1089/cyber.2013.0044 23617225

[B7] Boer H. Westhoff Y. (2006). The role of positive and negative signaling communication by strong and weak ties in the shaping of safe sex subjective norms of adolescents in South Africa. *Commun. Theor.* 16 75–90. 10.1111/j.1468-2885.2006.00006.x

[B8] Boerman S. C. (2019). The effects of the standardized Instagram disclosure for micro-and meso-influencers. *Comput. Hum. Behav.* 103 199–207. 10.1016/j.chb.2019.09.015

[B9] Boerman S. C. Van Reijmersdal E. A. Neijens P. C. (2015). Using eye tracking to understand the effects of brand placement disclosure types in television programs. *J. Advert.* 44 196–207. 10.1080/00913367.2014.967423

[B10] Boyle S. C. LaBrie J. W. Froidevaux N. M. Witkovic Y. D. (2016). Different digital paths to the keg? How exposure to peers’ alcohol-related social media content influences drinking among male and female first-year college students. *Addict. Behav.* 57 21–29. 10.1016/j.addbeh.2016.01.011 26835604 PMC5098897

[B11] Cialdini R. B. Trost M. R. (1999). “Social influence: social norms, conformity and compliance,” in *The Handbook of Social Psychology*, eds Gilbert D. T. Fiske S. T. Lindzey G. (New York, NY: McGraw-Hill), 151–192.

[B12] Coates A. E. Hardman C. A. Halford J. C. Christiansen P. Boyland E. J. (2019). Social media influencer marketing and children’s food intake: a randomized trial. *Pediatrics* 143:e20182554. 10.1542/peds.2018-2554 30833297

[B13] Collins A. M. Loftus E. F. (1975). A spreading-activation theory of semantic processing. *Psychol. Rev.* 82:407 10.1037//0033-295x.82.6.407

[B14] Curtis B. L. Lookatch S. J. Ramo D. E. McKay J. R. Feinn R. S. Kranzler H. R. (2018). Meta-analysis of the association of alcohol-related social media use with alcohol consumption and alcohol-related problems in adolescents and young adults. *Alcohol. Clin. Exp. Res.* 42 978–986. 10.1111/acer.13642 29786874 PMC5984178

[B15] Davoren M. P. Demant J. Shiely F. Perry I. J. (2016). Alcohol consumption among university students in Ireland and the United Kingdom from 2002 to 2014: a systematic review. *BMC Public Health* 16:173. 10.1186/s12889-016-2843-1 26895824 PMC4759952

[B16] de Vries L. Gensler S. Leeflang P. S. (2012). Popularity of brand posts on brand fan pages: an investigation of the effects of social media marketing. *J. Interact. Mark.* 26 83–91. 10.1016/j.intmar.2012.01.003

[B17] Domingues Aguiar T. van Reijmersdal E. A. (2018). *Influencer Marketing.* Amsterdam: SWOCC.

[B18] Erdogan B. Z. (1999). Celebrity endorsement: a literature review. *J. Mark. Manag.* 15 291–314. 10.1362/026725799784870379

[B19] Forsey C. (2019). *The Ultimate List of Instagram Influencers In Every Industry.* Available at: https://blog.hubspot.com/marketing/instagram-influencers (accessed October 22, 2019).

[B20] Geusens F. Beullens K. (2016). The association between social networking sites and alcohol abuse among belgian adolescents. *J. Media Psychol.* 30 207–216. 10.1027/1864-1105/a000196

[B21] Geusens F. Bigman-Galimore C. A. Beullens K. (2019). A cross-cultural comparison of the processes underlying the associations between sharing of and exposure to alcohol references and drinking intentions. *New Media Soc.* 22 49–69.

[B22] Global Advertising Lawyers Alliance (2011). *Alcohol Advertising: A Global Legal Perspective.* Available at: http://iogt.org/wp-content/uploads/2015/03/Alcohol-Advertising_A-Global-Legal-Perspective.pdf (accessed August 28, 2019).

[B23] Gross J. Wangenheim F. V. (2018). The big four of influencer marketing. A typology of influencers. *Mark. Rev. St. Gallen* 2 30–38.

[B24] Hashoff (2017). *Influencer Marketer. A #Hashoffstate of The Union Report.* Available at: https://www.slideshare.net/FilippPaster/hashoff-instagram-dominates-influencer-marketing-report (accessed August 29, 2019).

[B25] Hendriks H. Gebhardt W. A. van den Putte B. (2017). Alcohol-related posts from young people on social networking sites: content and motivations. *Cyberpsychol. Behav. Soc. Netw.* 20 428–435. 10.1089/cyber.2016.0640 28650223

[B26] Hendriks H. van den Putte B. Gebhardt W. A. (2018a). Alcoholposts on social networking sites: the alcoholpost-typology. *Cyberpsychol. Behav. Soc. Netw.* 21 463–467. 10.1089/cyber.2017.0729 29995528

[B27] Hendriks H. van den Putte B. Gebhardt W. A. Moreno M. A. (2018b). Social drinking on social media: content analysis of the social aspects of alcohol-related posts on facebook and instagram. *J. Med. Internet Res.* 20:e226. 10.2196/jmir.9355 29934290 PMC6035352

[B28] Hingson R. W. Zha W. (2009). Age of drinking onset, alcohol use disorders, frequent heavy drinking, and unintentionally injuring oneself and others after drinking. *Pediatrics* 123 1477–1484. 10.1542/peds.2008-2176 19482757

[B29] Hughes K. Anderson Z. Morleo M. Bellis M. A. (2008). Alcohol, nightlife and violence: the relative contributions of drinking before and during nights out to negative health and criminal justice outcomes. *Addiction* 103 60–65. 10.1111/j.1360-0443.2007.02030.x 17996008

[B30] Influencity (2018). *Types of Influencers and Their Characteristics.* Available at: https://influencity.com/blog/en/types-of-influencers-and-their-characteristics/ (accessed October 22, 2019).

[B31] Jackson M. C. Hastings G. Wheeler C. Eadie D. MacKintosh A. M. (2000). Marketing alcohol to young people: implications for industry regulation and research policy. *Addiction* 95 597–608. 10.1080/09652140020013809 11218354

[B32] Jernigan D. H. Padon A. Ross C. Borzekowski D. (2017). Self-reported youth and adult exposure to alcohol marketing in traditional and digital media: results of a pilot survey. *Alcohol. Clin. Exp. Res.* 41 618–625. 10.1111/acer.13331 28219114

[B33] Jones S. C. Thom J. A. Davoren S. Barrie L. (2014). Internet filters and entry pages do not protect children from online alcohol marketing. *J. Public Health Policy* 35 75–90. 10.1057/jphp.2013.46 24257629

[B34] Katz E. (1957). The two-step flow of communication: an up-to-date report of anhypothesis. *Public Opin. Q.* 21 61–78.

[B35] Knitter M. (2019). *Recent Case Law Clarifies Influencer Marketing and Labeling Requirements in Germany.* Available at: https://www.inta.org/INTABulletin/Pages/Influencers_and_Labelling_in_Germany_7401.aspx (accessed November 20, 2019).

[B36] Knoll J. Matthes J. (2017). The effectiveness of celebrity endorsements: a meta-analysis. *J. Acad. Mark. Sci.* 45 55–75. 10.1007/s11747-016-0503-8

[B37] Lazarsfeld P. F. Berelson B. Gaudet H. (1944). *The People’s Choice: How Thevoter Makes Up His Mind in a Presidential Campaign.* New York, NY: Columbia University.

[B38] Lord K. R. Putrevu S. (2009). Informational and transformational responses to celebrity endorsements. *J. Curr. Issues Res. Advert.* 31 1–13. 10.1080/10641734.2009.10505253

[B39] Lou C. Yuan S. (2019). Influencer marketing: how message value and credibility affect consumer trust of branded content on social media. *J. Interact. Advert.* 19 58–73. 10.1080/15252019.2018.1533501

[B40] McCracken G. (1986). Culture and consumption: a theoretical account of the structure and movement of the cultural meaning of consumer goods. *J. Consum. Res.* 13 71–84.

[B41] Moreno M. A. Briner L. R. Williams A. Brockman L. Walker L. Christakis D. A. (2010). A content analysis of displayed alcohol references on a social networking web site. *J. Adolesc. Health* 47 168–175. 10.1016/j.jadohealth.2010.01.001 20638009 PMC2907358

[B42] Moreno M. A. Christakis D. A. Egan K. G. Brockman L. N. Becker T. (2012). Associations between displayed alcohol references on Facebook and problem drinking among college students. *Arch. Pediatr. Adolesc. Med.* 166 157–163. 10.1001/archpediatrics.2011.180 21969360 PMC3266463

[B43] Nationale Drug Monitor (2017). *Jaarbericht 2017.* Utrecht: Trimbos Institute.

[B44] Ofcom (2017). *Children and Parents: Media Use and Attitudes Report.* Available at: https://www.ofcom.org.uk/__data/assets/pdf_file/0020/108182/children-parents-media-use-attitudes-2017.pdf (accessed September 11, 2019).

[B45] Perkins H. W. Berkowitz A. D. (1986). Perceiving the community norms of alcohol use among students: some research implications for campus alcohol education programming. *Int. J. Addict.* 21 961–976. 10.3109/10826088609077249 3793315

[B46] Rehm J. Room R. Graham K. Monteiro M. Gmel G. Sempos C. T. (2003). The relationship of average volume of alcohol consumption and patterns of drinking to burden of disease: an overview. *Addiction* 98 1209–1228. 10.1046/j.1360-0443.2003.00467.x 12930209

[B47] Rogers E. M. (1983). *Diffusion of Innovations*, 3rd Edn, New York, NY: FreePress.

[B48] Ruijs J. (2018). *Medialogica: Alcoholreclame op Instagram.* Available at: https://www.human.nl/medialogica/kijk/online/alcoholreclame.html (accessed September 1, 2019).

[B49] Silveri M. M. (2012). Adolescent brain development and underage drinking in the United States: identifying risks of alcohol use in college populations. *Harvard Rev. Psychiatry* 20 189–200. 10.3109/10673229.2012.714642 22894728 PMC4669962

[B50] Stevens G. Van Dorsselaer S. Boer M. De Roos S. Duinhof E. De Looze M. (2017). *HBSC 2017: Gezondheid en Welzijn Van Jongeren in Nederland.* Utrecht: Universiteit Utrecht.

[B51] Thomasius R. (2018). *Whatsapp, Instagram and Co. - so Addictive is Social Media.* Available at: https://www.saferinternet.at/fileadmin/redakteure/Footer/Studien/dak-studie-social-media-nutzung-1968596.pdf (accessed August 26, 2019).

[B52] Thompson C. M. Romo L. K. (2016). College students’ drinking and posting about alcohol: forwarding a model of motivations, behaviors, and consequences. *J. Health Commun.* 21 688–695. 10.1080/10810730.2016.1153763 27186824

[B53] Van den Putte B. Yzer M. Southwell B. G. de Bruijn G. J. Willemsen M. C. (2011). Interpersonal communication as an indirect pathway for the effect of antismoking media content on smoking cessation. *J. Health Commun.* 16 470–485. 10.1080/10810730.2010.546487 21337250

[B54] Van Dorsselaer S. V. Tuithof M. Verdurmen J. Spit M. Van Laar M. Monshouwer K. (2016). *Jeugd en Riskant Gedrag 2015. Kerngegevens Uit Het Peilstationonderzoek Scholieren.* Utrecht: Trimbos Institute.

[B55] World Health Organization [WHO] (2006). *Framework for Alcohol Policy in the WHO European Region.* Geneva: World Health Organization.

